# Refocusing Algorithm for Correlation Plenoptic Imaging

**DOI:** 10.3390/s22176665

**Published:** 2022-09-03

**Authors:** Gianlorenzo Massaro, Francesco V. Pepe, Milena D’Angelo

**Affiliations:** 1 Dipartimento Interateneo di Fisica, Università degli Studi di Bari, 70125 Bari, Italy; 2 Istituto Nazionale di Fisica Nucleare, Sezione di Bari, 70125 Bari, Italy

**Keywords:** light-field imaging, quantum imaging, correlation imaging, 3D imaging

## Abstract

Correlation plenoptic imaging (CPI) is a technique capable of acquiring the *light field* emerging from a scene of interest, namely, the combined information of intensity and propagation direction of light. This is achieved by evaluating correlations between the photon numbers measured by two high-resolution detectors. Volumetric information about the object of interest is decoded, through data analysis, from the measured four-dimensional correlation function. In this paper, we investigate the relevant aspects of the refocusing algorithm, a post-processing method that isolates the image of a selected transverse plane within the 3D scene, once applied to the correlation function. In particular, we aim at bridging the gap between existing literature, which only deals with refocusing algorithms in case of continuous coordinates, and the experimental reality, in which the correlation function is available as a discrete quantity defined on the sensors pixels.

## 1. Introduction

A plenoptic (or light-field) device can measure, at the same time, both the light intensity distribution and the propagation direction of the light rays that come out of a scene of interest [1,2,3,4,5]. The latter kind of information, which is not available to conventional imaging, endows a plenoptic device with three-dimensional imaging capabilities, allowing one to “refocus“, in post-processing, details of the scene that were out of focus at the moment of the capture. The operation of refocusing, if applied repeatedly to retrieve a stack of many axial planes of the sample, allows one to reconstruct a three-dimensional “cube“ that is very similar to the one obtained by 3D techniques relying on *z*-scanning [6,7], without the obvious disadvantages stemming from having an experimental device with moving parts. Plenoptic imaging is currently used in a variety of different applications such as photography [2,3], microscopy [8], real-time imaging of neuronal activity [9], and wavefront sensing [10]. Conventionally, the directional information needed for refocusing is obtained by inserting an array of micro-lenses within the optical setup; the micro-lenses, however, impose a trade-off between the 3D capabilities of the device and the maximum resolution that is attained [11]. Such tradeoff can be overcome through a recent alternative approach, which entirely avoids the use of microlenses. This method, called *correlation plenoptic imaging* (CPI), retrieves plenoptic information by measuring correlations between the photon number (i.e., intensity) fluctuations measured by two separate high-resolution sensors [12,13,14]. Combined spatial and directional information is retrieved with the maximal optical resolution determined by wave-optics; the resolution on the focused plane is thus only limited by the lens aperture [13] and the DOF extension wider than in conventional plenoptic imaging [15,16]. Remarkably, the resolution limits of images refocused by CPI are practically independent of the numerical aperture of the imaging system; this provides an improvement with respect to both standard imaging and traditional plenoptic imaging, at arbitrarily large distances from the focused plane [16]. Though the main embodiment of CPI have so far been designed to exploit the correlations of chaotic light [16,17], the technique is versatile enough to enable working also with the purely quantum correlations of entangled photon pairs [18], provided the setup is adequately modified [19].

Despite the differences in both the setup architecture and in the performances of traditional plenoptic imaging and CPI, the working principle of refocusing is unchanged: a collection of sub-images, representing the scene as viewed from different points of view (or different angles), must be realigned and integrated in order to get the refocused image of a specific plane. In both cases, the finite size and the discrete structure of sensors entail deviations from the ideal case (formulated in a continuous space); summing images with different fields of view and pixel centering are further issues than needs to be addressed. These problems have already been taken on in the context of traditional plenoptic imaging (see, e.g., Refs. [20,21]). However, correlation imaging brings along peculiar issues: the interplay between light propagating along the two correlated beams, and the crucial role of coherence acquired during propagation. These specific aspects require a dedicated analysis, as they are not encompassed in the research conducted for traditional plenoptic devices. Moreover, the higher versatility of CPI makes it necessary to develop a comprehensive theory, in which the problem of refocusing is tackled in the most general case (encompassing even the intricate algorithms that arise in so called correlation plenoptic imaging between arbitrary planes [15]).

In this work, we shall address these points and analyze the steps needed to obtain the refocused image from the measured correlation function. Particular attention will be devoted to the algorithmic complications that arise from its discrete nature.

## 2. Materials and Methods

In CPI, the refocused images are obtained by applying a refocusing algorithm to the measured correlation function

(1) $$\Gamma^{\left(2\right)}\left(r_{a},r_{b}\right)=\langle I_{A}\left(r_{a}\right)I_{B}\left(r_{b}\right)\rangle -\kappa \langle I_{A}\left(r_{a}\right)\rangle \langle I_{B}\left(r_{b}\right)\rangle ,$$

where $I_{A}$ and $I_{B}$ are the instantaneous light intensities measured by the two detectors, with 2D coordinates $r_{a}=\left(x_{a},y_{a}\right)$ and $r_{b}=\left(x_{b},y_{b}\right)$, respectively, and $\langle X\rangle$ denotes the expectation value of the observable *X*; κ is a constant that can either be 1 or 0, depending on the statistical properties of the illumination source. A detailed discussion about how the correlation function is calculated from the experimental data is reported in Ref. [22]. In the same paper, is is also shown that, although the correlation function of Equation (1) is a four-dimensional quantity, most of its properties still emerge by considering a simplified two-dimensional scenario, in which the two detectors are one-dimensional. Throughout the rest of the paper, the correlation function will be considered as a 2D quantity, with the two coordinates $x_{a}$ and $x_{b}$ defined on 1D detectors that will be called $D_{A}$ and $D_{B}$, respectively. Given the correlation function, the object detail at coordinate *x* on the transverse plane of the sample is reconstructed through the line integral

(2) $$\Sigma_{z}\left(x\right)=\int_{\gamma_{z}\left(x\right)}\Gamma^{\left(2\right)}\left(x_{a},x_{b}\right)ds.$$

**Figure 1:**
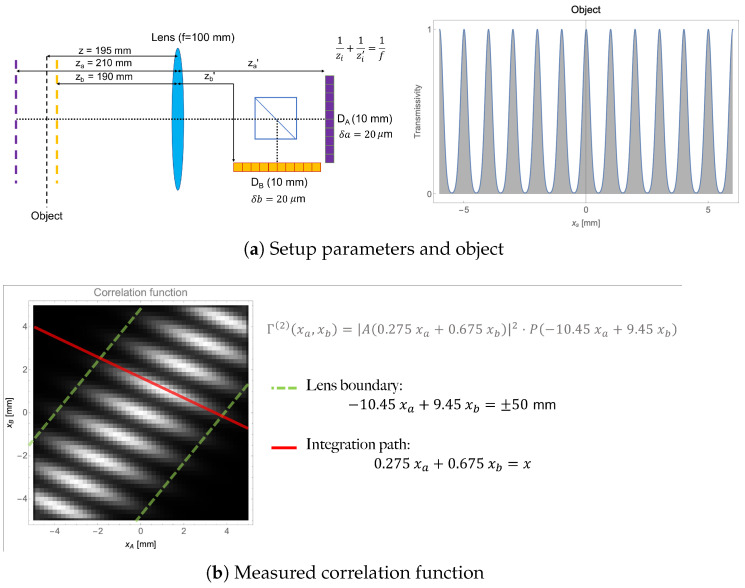
Simulation of a correlation function in the experimental setup shown in panel (**a**). The object is composed of a set of equally spaced 200μm-wide gaussian slits, centered 1mm apart from each other. Panel (**b**) shows the two-dimensional quantity that is reconstructed by measuring intensity correlations in the setup shown in panel (**a**). Since the detectors $D_{A}$ and $D_{B}$ are identical strips of 50 pixels each, the result of the measurement is a square matrix of 50×50 pixels. However, the optical distances involved and the finite radius of the lens (here fixed at 50mm), prohibits information to be contained in the pixels outside of the two dashed green lines [22]. The red segment, spanning the whole photosensitive area identified by the two detectors, represents the integration path for recovering the information at coordinate *x* in the object plane. The simulated correlation function is obtained by applying Equation (4) of Ref. [22] to the known object shape *A*; the function Φ is defined by the optical distances and components involved. Discretization is then imposed by integrating over the pixel size.

The whole object is reconstructed by repeating this integral for all *x* coordinates within the field of view (FOV). The curve $\gamma_{z}\left(x\right)$ is a line in the $\left(x_{a},x_{b}\right)$ plane defined by the detectors, whose slope depends solely on the the longitudinal position *z* of the sample along the optical axis, while the intercept depends on the specific *x* coordinate [22]. The possibility to implement a refocusing algorithm can be easily recognized by considering the form Equation (1) assumes in the geometrical (ray-optics) approximation, that is

(3) $$\Gamma^{\left(2\right)}\left(x_{a},x_{b}\right)∼{\left|A\left(\alpha \left(z\right)x_{a}+\beta \left(z\right)x_{b}\right)\right|}^{2}\;P\left(p_{1}x_{a}+p_{2}x_{b}\right)\;\chi_{A}\left(x_{a}\right)\;\chi_{B}\left(x_{b}\right),$$

where A(x) is the sample intensity profile (or field transmissivity, depending on whether the object is placed in both optical paths or in only one of them [22]), P(x) is the dominant limiting aperture within the optical setup, $\chi_{A}\left(x_{a}\right)$ and $\chi_{B}\left(x_{b}\right)$ are the characteristic functions of the detectors [22]. Both P and χ are vanishing outside of the transmissive area of the main iris, for P, and the photosensitive area of the detectors for $\chi_{A,B}$. The form in which the intensity profile of the sample A appears in Equation (3) makes it easy to understand refocusing: the sample detail at coordinate *x* is “spread” along a line of equation $\alpha \left(z\right)x_{a}+\beta \left(z\right)x_{b}=x$ in the $\left(x_{a},x_{b}\right)$ plane defined by the detectors, the value of ${\left|A(x)\right|}^{2}$ can thus be recovered by applying Equation (2). Figure 1 shows the simulation of the correlation function (Figure 1b) measured by a single-lens CPI configuration (Figure 1a). In this particular scheme, detectors imaging two planes that are slightly displaced from one another along the optical axis are correlated ($z_{a}\neq z_{b}$). If the detectors and the optical paths leading to them are considered separately, the device behaves as two different single-lens imaging systems, each retrieving the image of a given plane, with the DOF and resolution defined by the lens; the object is placed out of the DOF of the two systems and thus cannot be resolved. However, if the light intensity impinging on the two detectors are measured, plenoptic information about the sample can be retrieved as from the equation reported in the left hand side of Figure 1b (see Equation (3)).

In the next section, we shall discuss how to apply the refocusing algorithm to the experimental correlation function, keeping into account the effect of the size of both the detectors and their pixels.

## 3. Results

As we have already mentioned, in order to obtain a complete reconstruction of the object, the line integral of Equation (2) must be performed for all coordinates *x* within the FOV. Since the intensity measurements are performed with photodetectors having a finite pixel size, the refocusing coordinate *x* spans the FOV in discrete steps that must depend on the pixel size and the other experimental parameters. Before dealing with such discretization, however, let us establish how the finite size of the detectors define the FOV of CPI.

### 3.1. Field of View

In conventional imaging, the FOV is given by the portion of the object that is imaged on the photosensor. If optical distortion and other artifacts such as vignetting are neglected, the FOV is essentially determined by the detector size alone, and does not depend on the size of the optical elements. As demonstrated in Ref. [22], the same is true in CPI, where the finite size of the optical components can alter other properties of the final images, but not the FOV. Still, since CPI is based on two detectors, the identification of the FOV is less trivial than in standard imaging. In fact, based on Equation (3), the combination of the two photosensitive surfaces of $D_{A}$ and $D_{B}$ is the rectangle of equation $\chi_{A}\left(x_{a}\right)\cdot \chi \left(x_{b}\right)\neq 0$ in the $\left(x_{a},x_{b}\right)$ plane. The FOV of CPI can thus be defined as the set of the *x* coordinates for which the line $\alpha \left(z\right)x_{a}+\beta \left(z\right)x_{b}=x$ intersects the rectangle defined by the detectors (see Figure 1b and, for further details, Figure 4a of Ref. [22]), and can be obtained as the difference between the maximum and the minimum values of *x* that produces a line in the $\left(x_{a},x_{b}\right)$ plane having non-null intersection with the photosensitive area. By indicating with $L_{i}=N_{i}\cdot \delta i$ the linear size of the detectors, with $N_{i}$ the number of pixels, δi the pixel size, and i=a,b, the FOV of CPI is

(4) $$\mathrm{FOV}\left(z\right)=\left|\alpha (z)\right|\delta a\cdot N_{A}+\left|\beta (z)\right|\delta b\cdot N_{B}.$$

A comparison with the FOV of conventional imaging is provided in Appendix A.

### 3.2. The Refocusing Transformation

When reconstructed from experimental data, the correlation function of Equation (1) is a $N_{A}\times N_{B}$ real matrix, whose rows correspond to pixels on $D_{A}$ and columns correspond to pixels on $D_{B}$. For adapting the refocusing integral of Equation (2) to such discrete and finite case correlation function, two issues need to be addressed:

- for any given integration line (i.e., for any fixed value of *x*), choosing the appropriate spacing between the points along the integration path to properly reconstruct the image;
- for *x* varying in the FOV defined in Equation (4), choosing the adequate spacing between neighbouring integration lines, which physically translates into the granular resolution of the refocusing process.

The intuitive idea that smaller steps along both the integration lines and the distance between lines lead to finer results is not really correct. In fact, the integration lines employed for image reconstruction run through points, in the $\left(x_{a},x_{b}\right)$ plane, which do not coincide with the discrete coordinates on which the correlation function is defined, but can rather assume any value, obtained through four-dimensional interpolation of the experimental dataset. During this interpolation stage, the correlation function should not be oversampled to avoid needlessly long computation time. On the other hand, also undersampling aimed at shortening the computation time must be avoided. One intuitive reason is that undersampling along the *x* direction would entail a loss of resolution, but also undersampling along the integration direction has less intuitive side-effects on the signal-to-noise ratio of the refocused image. In fact, although the integration lines contain, in principle, “copies” of the same object detail located at coordinate *x*, the availability of a large number of copies and the choice of using all of them for refocusing offers the possibility to maximize the signal-to-noise ratio of the final image [16].

In addition, due to the finite size of the detectors, integration along the lines $\gamma_{z}\left(x\right)$ in Equation (2) can only occur on the segments intercepted by the lines on the photosensitive rectangle in the $\left(x_{a},x_{b}\right)$ plane (Figure 1b). The length of these segments, the integration extremes, and the number of resampling steps, depend on both *z*, through the line slope, and on the refocusing coordinate *x*. By choosing the following *t*-parametrization for the integration segment,

(5) $$\gamma_{z}\left(x,t\right):\left\{\begin{array}{cc} \begin{array}{c} x_{a}=x_{a}\left(x,t\right)\\ y_{a}=y_{a}\left(x,t\right) \end{array} & t\in \left[a_{z}\left(x\right),b_{z}\left(x\right)\right] \end{array}\right.,$$

Equation (2) can be rewritten as a Riemann integral

(6) $$\Sigma_{z}\left(x\right)=\int_{a_{z}\left(x\right)}^{b_{z}\left(x\right)}\Gamma^{\left(2\right)}\left(x_{a}\left(x,t\right),x_{b}\left(x,t\right)\right)\sqrt{{\left(\frac{\partial x_{a}}{\partial t}\right)}^{2}+{\left(\frac{\partial x_{b}}{\partial t}\right)}^{2}}dt,$$

whose extremes depend on both *z* and the particular refocusing point *x*.

It is worth noticing that, although Equation (5) parametrizes $\gamma_{z}\left(x\right)$ through the single parameter *t*, it still carries an implicit dependence on the variable *x*. Therefore, Equation (5) can be regarded as a coordinate transformation from the $\left(x_{a},x_{b}\right)$ plane to a new (x,t) plane, in which the first coordinate is the “refocusing” coordinate and the second one is an integration coordinate. In the transformed plane, all the contributions in the correlation function related to the same object point are lined up along the vertical. In fact, if we consider the transformed function $\Gamma_{\mathrm{trans}}^{\left(2\right)}\left(x,t\right)=\Gamma^{\left(2\right)}\left(x_{a}\left(x,t\right),x_{b}\left(x,t\right)\right)\sqrt{{\left(\partial_{t}x_{a}\right)}^{2}+{\left(\partial_{t}x_{b}\right)}^{2}}$, we see that Equation (6) is just an integration on vertical segments of the function $\Gamma_{\mathrm{trans}}^{\left(2\right)}$. The coordinate transformation in Equation (5) shall thus be called a *refocusing transformation*. Since it stems from the parametrization of a line, which can be obtained in infinite equivalent ways, many equivalent refocusing transformations can also be chosen. For simplicity, let us consider a transformation that is linear in both *x* and *t*, so that the square root term in Equation (6) becomes a constant, common to all refocusing points *x*, and can be disregarded. In this case Equation (5) can be conveniently written in the matrix form

(7) $$\left(\begin{array}{c} x_{a}\\ x_{b} \end{array}\right)=A\left(\begin{array}{c} x\\ t \end{array}\right),$$

where *A* is a 2×2 matrix, whose coefficients of *B* are determined by imposing that it is a refocusing transformation. This implies, first of all, that the transformation parametrizes the line $x=\alpha \left(z\right)x_{a}+\beta \left(z\right)x_{b}$; this line represents a constraint on the first row of a matrix *B*, involved in the inverse transformation

(8) $$\left(\begin{array}{c} x\\ t \end{array}\right)=B\left(\begin{array}{c} x_{a}\\ x_{b} \end{array}\right),$$

where $B_{11}=\alpha \left(z\right)$ and $B_{12}=\beta \left(z\right)$. In addition, since the refocusing matrix is the inverse of *B* ($A=B^{-1}$), as can be clearly seen by comparing Equations (7) and (8), the second row of *B* must be chosen so as to guarantee that the matrix is non-singular and *A* is well-defined.

#### 3.2.1. Integration Extremes

With the considerations above, when a linear refocusing transformation is chosen, the refocusing algorithm in Equation (6) can be expressed as

(9) $$\Sigma_{z}\left(x\right)=\int_{a_{z}\left(x\right)}^{b_{z}\left(x\right)}\Gamma^{\left(2\right)}\left[A\left(\begin{array}{c} x\\ t \end{array}\right)\right]dt.$$

**Figure 2:**
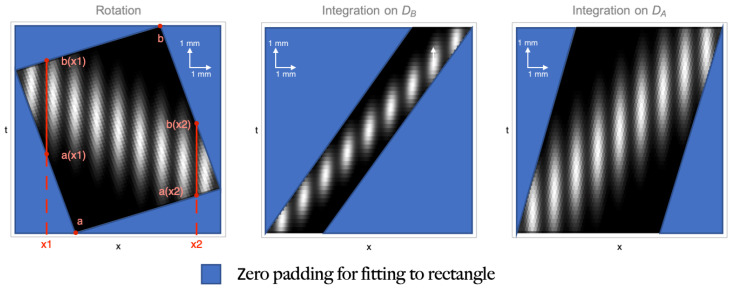
Three possible refocusing transformations applied to the correlation function of Figure 1b.

We are now interested in finding an expression for the integration extremes $a_{z}\left(x\right)$ and $b_{z}\left(x\right)$, that are expected to depend on the refocusing transformation as well. In the $\left(x_{a},x_{b}\right)$ plane, the boundary to the integration region is the rectangle enclosed by the four lines $x_{a}=\pm N_{A}/2\cdot \delta a$ and $x_{b}=\pm N_{B}/2\cdot \delta b$; the equations of these lines in the transformed (x,t) plane will set the vertical boundaries to the Riemann integral in Equation (9). Thus, the boundaries to the integration region are determined by the four lines

(10) $$\begin{array}{cc} r_{1}\left(x,t\right): & A_{11}\;x+A_{12}\;t=+N_{A}/2\cdot \delta a\\ r_{2}\left(x,t\right): & A_{11}\;x+A_{12}\;t=-N_{A}/2\cdot \delta a\\ s_{1}\left(x,t\right): & A_{21}\;x+A_{22}\;t=+N_{B}/2\cdot \delta b\\ s_{2}\left(x,t\right): & A_{21}\;x+A_{22}\;t=-N_{B}/2\cdot \delta b \end{array},$$

where $A_{ij}$ are the coefficients of the 2×2 matrix *A*. In fact, the four lines are those that define the boundaries of the detection area in Figure 2. Since $r_{1}\|r_{2}$ and $s_{1}\|s_{2}$, it is always possible to determine which one of the two lines in the pairs is greater than the other at any given *x*, so that one can define the two lines defining the lower boundary of the integration area as $r_{↓}\left(x,t\right)=\min\left\{r_{1},r_{2}\right\}$ and $s_{↓}\left(x,t\right)=\min\left\{s_{1},s_{2}\right\}$, and the same for the upper boundary, namely, $r_{↑}\left(x,t\right)=\max\left\{r_{1},r_{2}\right\}$ and $s_{↑}\left(x,t\right)=\max\left\{s_{1},s_{2}\right\}$. Once the upper and lower lines are identified, the integration extremes are easily defined. In fact, the lower extreme is the greatest between the two values that the lines $r_{↑}$ and $s_{↑}$ assume at the given *x* of interest, namely,

(11) $$a_{z}\left(x\right)=\max\left\{t_{↓}|r_{↓}\left(x,t_{↓}\right)=0\;\mathrm{or}\;s_{↓}\left(x,t_{↓}\right)=0\right\}$$

and, analogously,

(12) $$b_{z}\left(x\right)=\min\left\{t_{↑}|r_{↑}\left(x,t_{↑}\right)=0\;\mathrm{or}\;s_{↑}\left(x,t_{↑}\right)=0\right\}.$$

For a better understanding of this reasoning, the left plot of Figure 2 shows how, for the given transformation, the integration extremes change with the considered coordinate *x*. For the sake of completeness, we should point out that this procedure for finding the integration extremes does not work when either $A_{12}=0$ or $A_{22}=0$ (lines parallel to the *t* axis). This two cases are actually trivial, both because the integration domain is completely defined by the other two lines, and because these two scenarios correspond to the object being at focus on either one of the two planes imaged by $D_{A}$ and $D_{B}$, so that the “refocused” image can be obtained by simply integrating on the other detector.

Figure 2 shows the equivalence of three different refocusing transformations, applied to the correlation function of Figure 1b. The corresponding matrices are, from left to right,

$$\begin{array}{ccccc} A_{\mathrm{rot}}=\left(\begin{array}{cc} \cos\theta & \sin\theta \\ -\sin\theta & \cos\theta \end{array}\right) & & A_{D_{B}}=\left(\begin{array}{cc} 3.64 & -2.45\\ 0 & 1 \end{array}\right) & & A_{D_{A}}=\left(\begin{array}{cc} 0 & 1\\ 1.48 & -0.41 \end{array}\right), \end{array}$$

where $\theta =-\arctan\left(0.41\right)$ is the slope of the integration paths; the coefficients are obtained for the experimental parameters in Figure 1b. All the transformations line up the details along the vertical direction; however, the first one does it through a simple rotation of the $\left(x_{a},x_{b}\right)$ plane, the second one by applying a shear parallel to $D_{A}$, and the third one through a shear along $D_{B}$. The transformations are equivalent in refocusing the object, but the three boundaries defined by the lines in Equation (10) are rather different, implying a significant difference between the integration extremes in Equation (9).

We have thus shown that, due to the finite size of the detectors and the transformation of the boundaries when the refocusing transformation is applied, the integration extremes are a function of the particular *x* coordinate that is chosen. However, there are cases in which keeping track of the *x* dependence is inconvenient: in fact, one can typically solve the integral of Equation (9) by

- either generating, for each *x*, the list of integration points that will contribute to that point, in which case it is not an issue to have a number of sampled points that varies with *x*
- or by applying the transformation to the correlation function and resampling it on a regular grid in the (x,t) plane, so that the refocused image is obtained by simply collapsing the columns of the refocused matrix (i.e., the *t* coordinate).

**Figure 3:**
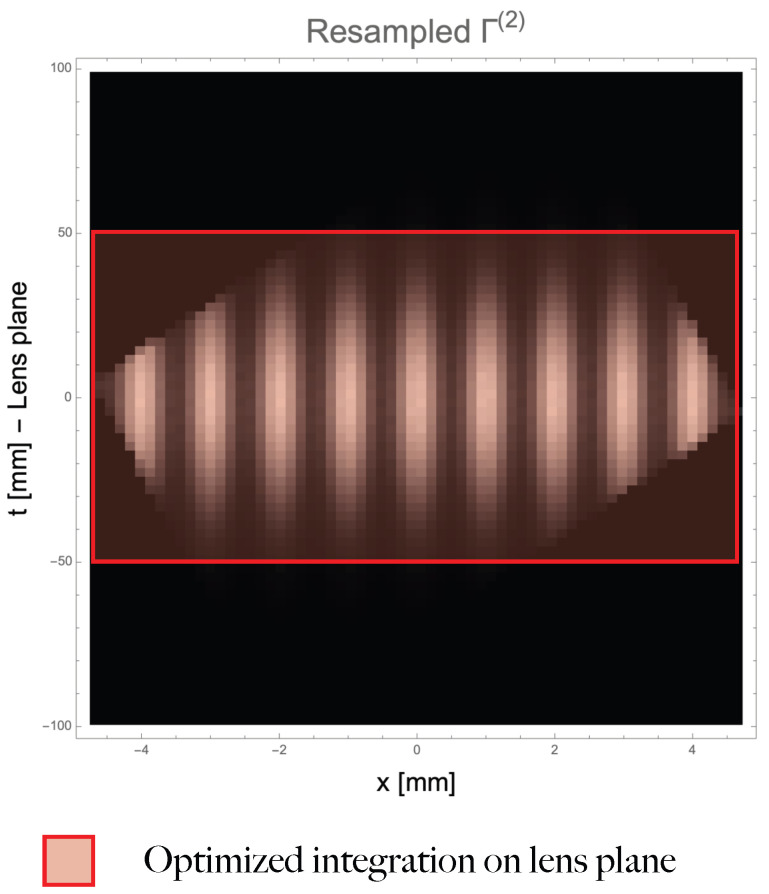
Resampling of the correlation function of Figure 1b on a regular grid in the (x,t) plane. The spacing between neighboring pixels is given by Equation (22) along the horizontal direction and (20) along the vertical.

The two operations above are, of course, completely equivalent from a mathematical point of view. However, working on regular grids is typically much more convenient computationally. This would entail choosing a fixed range $\left[a_{z},b_{z}\right]$ for the integration extremes, which, to avoid information loss, is determined by

(13) $$\begin{array}{cccc} a_{z}=\min_{x\in \mathrm{FOV}}\left\{a_{z}\left(x\right)\right\} & \; & & b_{z}=\max_{x\in \mathrm{FOV}}\left\{b_{z}\left(x\right)\right\} \end{array}$$

(see Figure 2). By doing so, the measured correlation function, that is intrinsically defined on a rectangle with sides $N_{A}\cdot \delta a$ and $N_{B}\cdot \delta b$, is transformed into a new rectangle having sides of length FOV(z) along *x*, and $b_{z}-a_{z}$ along *t*, as in Figure 3. Notice that, although the choice of working with a rectangular area might be convenient from a computational point of view, it is surely inconvenient from the point of view of memory management, since substantial zero-padding is required to fill-up points on which the correlation function is not natively defined (Figure 2). As demonstrated in Appendix B, the maximum integration range is given by

(14) $$b_{z}-a_{z}=\left|c\right|N_{A}\delta a+\left|d\right|N_{B}\delta b,$$

where *c* and *d* are the coefficients of the second row of the inverse refocusing matrix $B=A^{-1}$, that can be chosen arbitrarily. From the Equation (14), we see that *c* and *d* play the same role on the *t* axis that the coefficients α and β do in determining the FOV of the *x* axis. This property can be exploited by taking advantage of their arbitrariness to “cut-off” uninteresting regions of the measured correlation function and speed up the refocusing process, as displayed in Figure 3.

#### 3.2.2. Optimized Integration Area

Depending on the particular CPI scheme and the features of the involved optical components, the area defined by the aperture P in the $\left(x_{a},x_{b}\right)$ plane (see Equation (3)) can be smaller than the one defined by the photosensitive area of the detectors $\chi_{A}\cdot \chi_{B}$. In those cases, the experimental correlation function contains many points that should be disregarded upon refocusing, since they only contribute to noise [22]. Let us suppose the aperture function $P(x_{p})$ is an iris with radius *ℓ*, centered on the optical axis of the system. In this case, the only relevant portion of the correlation function is the one in which $-\ell \leq p_{1}x_{a}+p_{2}x_{b}\leq +\ell$. As long as the experimental parameters are known, this is easily taken into account by exploiting the degree of freedom on the second line of the $A^{-1}$ matrix and by choosing $c=p_{1}$ and $d=p_{2}$. In these conditions, the refocusing formula reads

(15) $$\Sigma_{z}\left(x\right)∼\int_{a_{z}\left(x\right)}^{b_{z}\left(x\right)}{\left|A\left(x\right)\right|}^{2}P\left(t\right)dt,$$

with the integrand being non-vanishing only for $\left|t\right|\leq \ell$. Hence, for all the object coordinates *x* for which the integration extremes defined by the detectors are larger than the limiting aperture, the integration path can be cut at ±ℓ, and the refocusing algorithm reduces to:

(16) $$\Sigma_{z,\mathrm{opt}}\left(x\right)=\int_{a_{z}^{\prime }\left(x\right)}^{b_{z}^{\prime }\left(x\right)}\Gamma^{\left(2\right)}\left[A\left(\begin{array}{c} x\\ t \end{array}\right)\right]dt,$$

with $a_{z}^{\prime }\left(x\right)=\min\left\{a_{z}\left(x\right),-l\right\}$ and $b_{z}^{\prime }\left(x\right)=\max\left\{b_{z}\left(x\right),+l\right\}$. Still, if one is interested in resampling the transformed correlation function in a rectangular domain, the integration extremes needs to be replaced with ±l, resulting in an integration length: $2l<\left|c\right|N_{A}\delta a+\left|d\right|N_{B}\delta b$, which enables sparing computation time.

### 3.3. Resampling of the Correlation Function

As the experimental correlation function is available in discrete “steps” (δa, δb), defined by the pixel pitch of the sensors, the refocusing process must also involve discrete steps in the process of image reconstruction. In this section, we deal with the aspects of the refocusing process related with discretization. We shall suppose that refocusing is performed in steps of δx along the refocusing direction, and of δt along the integration direction. The integral of Equation (9) thus entails resampling the correlation function in steps of δx, along the horizontal direction, and δt, along the vertical. The refocusing process for a given object coordinate *x* thus becomes

(17) $$\Sigma_{z,\mathrm{disc}}\left(x\right)=\sum_{i=0}^{N_{z}\left(x\right)-1}\Gamma_{\mathrm{trans}}^{\left(2\right)}\left(x,a_{z}\left(x\right)+i\cdot \delta t\right),$$

where N(x) is the closest integer to $(b_{z}\left(x\right)-a_{z}\left(x\right))/\delta t$. This operation must be repeated by sweeping the whole FOV in steps of δx, namely, assuming $x_{0}$ is the lower bound of the FOV, for $x=x_{0}+j\cdot \delta x$ with $j=0,\ldots ,M_{z}-1$, where $M_{z}$ is the closest integer to FOV(z)/δx.

The choice of the sampling steps must at least take into account that the transformed correlation function can only be resampled in its non-zero area, and that both undersampling and oversampling should be avoided. The most simple solution is to choose δx and δt such that the number of points for refocusing is approximately equal to the initial points within the relevant area, namely $\sum_{j=0}^{M_{z}-1}N_{z}\cdot \left(x_{0}+j\cdot \delta x\right)≃N_{A}\cdot N_{B}$. However, a more rigorous and effective solution consists in imposing a requirement on the point density rather than on the total number of points; this is performed by transforming each “unit” cell of area δa·δb, in the original plane, into a unit cell of area δx·δt, in the transformed plane, that contains the same number of experimental points (one). Unlike the condition on the total number of points, this condition has the advantage of holding also when the number of points is modified by either zero-padding or by cutting uninteresting parts of the function.

We shall now determine the δx and δt that are obtained by choosing that a single point in the measured space is mapped into a single point in the transformed space. To do so, rather than considering the transformations *A* and *B* that transform coordinates from the detector and the refocused planes and vice versa, we consider the matrices $A^{\prime }$ and $B^{\prime }$, mapping pixels indices, in place of the coordinates, on the refocused plane, and vice versa. The matrix $B^{\prime }$ is obtained by simply including the pixel size inside of the coefficients

(18) $$B^{\prime }=\left(\begin{array}{cc} \delta a\;\alpha (z) & \delta b\;\beta (z)\\ \delta a\;c & \delta b\;d \end{array}\right);$$

$A^{\prime }$ is given by its inverse. By doing this, $B^{\prime }$ maps the integer coordinates $n_{A}$ and $n_{B}$ in the discrete space of pixel indices onto the $\left(x,t\right)$ plane, with $n_{I}=1,\ldots ,N_{I}$, I=A,B. This is aimed at normalizing the cell area in the detector plane to unity, and making sure that the “weight” of pixels is kept into account when applying the transformation to and from the refocused plane. To understand how this is useful, let us start by calculating δt. To do so, we consider that a refocusing transformation satisfies a condition on the integration direction, that is given by the coefficients α(z) and β(z). This implies that the $B^{\prime }$ matrix transforms a vector oriented along the integration direction into a vector having only the *t* component in the (x,t) plane. This property can be used to impose a condition on δt. In fact, since we want an integration step in the transformed plane to have the same “weight” as in the original plane, we must impose that a vector having norm $∥\delta t∥$ and oriented along the vertical be transformed into a unit-vector in the $\left(n_{A},n_{B}\right)$ plane. That means requiring

(19) $$∥A^{\prime }\left(\begin{array}{c} 0\\ \delta t \end{array}\right)∥=1.$$

This condition is an equation in δt and binds it to the coefficients of the refocusing matrix *A* in Equation (7), or, alternatively, to the coefficients of its inverse *B*. The solution of this equation is

(20) $$\delta t=\frac{1}{\sqrt{{\left(\frac{a_{2}}{\delta a}\right)}^{2}+{\left(\frac{a_{4}}{\delta b}\right)}^{2}}}=\frac{\left|\det B\right|}{\sqrt{{\left(\frac{\beta (z)}{\delta a}\right)}^{2}+{\left(\frac{\alpha (z)}{\delta b}\right)}^{2}}}.$$

Now, to determine δx we impose the condition that the point density is conserved by the transformation $B^{\prime }$. To do this, we must impose that $B^{\prime }$ transforms the unit cell of the $\left(n_{A},n_{B}\right)$ plane into a cell having area δx·δt in the transformed space. This is performed by transforming the canonical basis of $\left(n_{A},n_{B}\right)$, evaluating the area of the cell it defines in the transformed space, and imposing that the point density is conserved, namely,

(21) $$∥B^{\prime }\left(\begin{array}{c} 1\\ 0 \end{array}\right)\times B^{\prime }\left(\begin{array}{c} 0\\ 1 \end{array}\right)∥=\left|\det B^{\prime }\right|=\delta a\cdot \delta b\left|\det B\right|=\delta x\cdot \delta t.$$

In this equation, the transformed unit cell area is evaluated by calculating the norm of the cross product between the transformed vectors. This defines an equation for δx, that, upon plugging-in the value of δt obtained from Equation (20), has solution

(22) $$\delta x=\sqrt{{\left(\alpha (z)\delta a\right)}^{2}+{\left(\beta (z)\delta b\right)}^{2}}.$$

As one might expect, the step on the refocusing axis depends only on the coefficients α and β, and not on the particular refocusing transformation that has been chosen (the coefficients *c* and *d*). Figure 3 shows the resampling of the correlation function in Figure 1b on a rectangular grid. The refocusing transformation and its inverse have been chosen as

$$\begin{array}{ccc} A=\left(\begin{array}{cc} 0.98 & -0.069\\ 1.08 & 0.028 \end{array}\right) & & A^{-1}=\left(\begin{array}{cc} 0.275 & 0.675\\ -10.45 & 9.45 \end{array}\right) \end{array}.$$

The parameters of the inverse matrix are exactly the four experimental parameters reported in Figure 1a. The first line of the inverse matrix is the one responsible for refocusing, while the second line becomes the vertical direction in the transformed plane. Thus, if the parameters of the second row are matched to those appearing in the limiting aperture, the aperture coordinates “line up” along the horizontal in the same fashion as the object features line up along the vertical because of the choice of the first line. Furthermore, the resampled function shown in Figure 3 has been adapted to fit a rectangle in the transformed plane, spanning the whole FOV along the *x* axis, and, the maximum integration range given by Equation (14) along *t*. The operation requires substantial zero-padding to extend the refocused function outside of the domain defined by Equation (10); however, as highlighted by the red rectangle, the same refocusing would be obtained by limiting the integration range to the lens size ([−50mm,+50mm]). Also, because of the particular transformation that has been chosen, limiting the resampling to the lens extension would also result in a convenient rectangular shape in the transformed plane.

## 4. Discussion

The purpose of this work is to offer a thorough understanding of the refocusing process, used to reconstruct an out-of-focus object from the correlation function measured by CPI. In particular, the aspects of refocusing related with the discrete nature of the measurement have been analyzed to bridge the gap between the theoretical refocusing algorithm in Equation (2), formulated in a continuous space, and the practical steps required to retrieve the final image from experimental data, captured by a pixel structure. To summarize the results of this work, we report below the procedure for extracting the refocused image from the measured data, which, interestingly, works in general for any CPI implementation.

1. The two parameters α and β of Equation (3) are estimated, as in Figure 1, through the knowledge of the experimental setup and the position *z* of the plane one wishes to refocus. If the lens aperture plays a significant role in limiting the non-zero area of the correlation function, also the parameters $p_{1,2}$ will be of interest, albeit independent of *z*.
2. The coefficients α and β, the number of pixels $N_{A}$ and $N_{B}$, and the pixels sizes δa and δb, determine the FOV of the refocused image through Equation (4). Typically, the pixel size is the same for $D_{A}$ and $D_{B}$, but sometimes binning is applied to either one of the two detectors; such binning must be taken into account when determining δa and δb.
3. An inverse refocusing matrix is chosen
  $$A^{-1}=\left(\begin{array}{cc} \alpha & \beta \\ c & d \end{array}\right),$$
  where α and β are the object parameters of Equation (3), and *c* and *d* are arbitrary numbers, whose value determine the amount of zero-padding needed to obtain a rectangular refocused function. When the limiting aperture is relevant in determining the non-zero area of the correlation function, the most convenient choice is: $p_{1}=c$, $p_{2}=d$.
4. To obtain a rectangular refocused function without loss of information, the resampling range along the vertical axis must be the one given in Equation (14). This operation, however, requires zero-padding. When integrating on the aperture ($p_{1}=c$, $p_{2}=d$), the range is given by the diameter of the aperture.
5. The resampling step along the horizontal direction of the refocused function is independent of the refocusing transformation and is given by Equation (22). The step along the vertical direction is transformation-dependent and is calculated through Equation (20).
6. The rectangular grid on which the refocused function must be resampled is obtained by spanning the FOV in discrete steps of δx and the integration range in discrete steps of δt.
7. The value of the resampled correlation function is obtained through interpolation of the experimental correlation function. In particular, to obtain the value of the transformed function in the point (x,t), the experimental function must be interpolated in the point $A^{-1}\left(\begin{array}{c} x\\ t \end{array}\right)$.
8. A rectangular refocused function is obtained when interpolation is applied to the whole rectangular domain in the transformed plane. The final image is obtained by summing together all the values within a column.
9. Often, to fully recover the object features with the correct relative intensity, the correction algorithm described in Equation (16) of Ref. [22] should be applied.

## Data Availability

Not applicable.

## Abbreviations

The following abbreviations are used in this manuscript:

CPI	Correlation plenoptic imaging
FOV	Field of view

## Appendix A. Field of View Comparison between CPI and Standard Imaging

We report the expression for the FOV of CPI as in Equation (4):

$$\mathrm{FOV}\left(z\right)=\left|\alpha (z)\right|\delta a\cdot N_{A}+\left|\beta (z)\right|\delta b\cdot N_{B}$$

and compare it with the FOV of standard imaging in the case of two specific CPI architectures.

### Appendix A.1. Correlation Light-Field Microscopy

Correlation light-field microscopy (CLM) is a CPI architecture oriented to microscopy, as in Ref. [16]. For this experimental realization,

$$\begin{array}{ccc} \alpha \left(z\right)=-\frac{z}{f}\frac{x_{a}}{M_{A}} & & \beta \left(z\right)=\left(1-\frac{z}{f}\right)\frac{x_{b}}{M_{B}} \end{array},$$

where *z* and *f* are the distance of the sample from the objective lens and the focal length of the objective, respectively, and $M_{A}$ and $M_{B}$ are the microscope magnification and the imaging magnification on $D_{B}$, respectively. Assuming detectors $D_{A}$ and $D_{B}$ are approximately the same size ($\delta aN_{A}≃\delta bN_{B}=L$), the FOV is

$${\mathrm{FOV}}_{\mathrm{CLM}}\left(z\right)=L\left[\frac{z}{f}\frac{1}{M_{A}}+\left|1-\frac{z}{f}\right|\frac{1}{M_{B}}\right],$$

whereas the FOV of a conventional microscope built with the same optical specifications is

$${\mathrm{FOV}}_{\mathrm{MIC}}=\frac{L}{M_{A}}.$$

Thus, the FOV of CLM is the same as a conventional microscope when the object is at focus (z=f), and becomes increasingly larger as the object is moved out of focus. If the FOV is expressed in the canonical form of the detector size over an equivalent magnification ${\mathrm{FOV}}_{\mathrm{CLM}}\left(z\right)=L/M\left(z\right)$, then the equivalent magnification of CPI is

(A1) $$M\left(z\right)=\frac{f}{z}{\left[\frac{1}{M_{A}}+\left|1-\frac{f}{z}\right|\frac{1}{M_{B}}\right]}^{-1},$$

which is always smaller than the native microscope magnification.

### Appendix A.2. CPI between Arbitrary Planes

In the single-lens configuration of CPI between arbitrary planes (Ref. [15]), the coefficients α and β are

$$\begin{array}{ccc} \alpha \left(z\right)=\frac{z-z_{b}}{z_{a}-z_{b}}\frac{1}{M_{A}}=\frac{c(z)}{M_{A}} & & \beta \left(z\right)=-\frac{z-z_{a}}{z_{a}-z_{b}}\frac{1}{M_{B}}=\frac{1-c(z)}{M_{B}} \end{array},$$

where *z* is the distance of the object from the lens, $z_{a}$ and $z_{b}$ are the distances from the lens of the two planes that are imaged onto $D_{A}$ and $D_{B}$, and

(A2) $$M_{i}=\frac{f}{z_{i}-f},$$

i=a,b are the two magnifications of the planes that are imaged on the detectors if the lens has focal length equal to *f*. Thus, in the approximation of same detector size,

$${\mathrm{FOV}}_{\mathrm{CLM}}\left(z\right)=L\left[\left|\frac{c(z)}{M_{A}}\right|+\left|\frac{1-c(z)}{M_{B}}\right|\right],$$

whereas the FOV of a single-lens focused imaging system would be

$${\mathrm{FOV}}_{\mathrm{STD}}\left(z\right)=\frac{L}{M_{0}\left(z\right)},$$

where $M_{0}\left(z\right)=f/\left(z-f\right)$. As in the previous case, when the object is at focus on either one of the two focused planes ($z=z_{a},z_{b}$), the FOV of CPI is the same as in standard imaging, while, for $z_{a}<z<z_{b}$ (if $z_{a}<z_{b}$) the FOV interpolates linearly the two FOVs at focus. For $z<z_{a}$ and $z>z_{b}$, the FOV increases linearly with the distance from the focused plane.

## Appendix B. Maximum Integration Range

In this appendix, we demonstrate Equation (14). As from the discussion preceding this formula in the text, the maximum integration range is given by the difference between maximum upper extreme $b_{z}$ and minimum lower extreme $a_{z}$. However, if one is only interested in the minimum (maximum) possible *t* coordinate on which the transformed correlation function is defined, an easier path can be followed instead of minimizing $a_{z}$ (and maximizing $b_{z}$). In fact, the extremal points of the transformed domain of the correlation function must be defined by the four vertices of the parallelogram defined by the lines $r_{1,2}$ and $s_{1,2}$. Since *A* is non-singular by definition, both *s*-lines intersect both *r*-lines, and viceversa, so that the four points are always well defined. Their coordinates are:

$$\begin{array}{cc} P_{11}: & \left(\alpha \left(z\right)\frac{N_{A}}{2}\delta a+\beta \left(z\right)\frac{N_{B}}{2}\delta b,c\frac{N_{A}}{2}\delta a+d\frac{N_{B}}{2}\delta b\right)\\ P_{12}: & \left(\alpha \left(z\right)\frac{N_{A}}{2}\delta a-\beta \left(z\right)\frac{N_{B}}{2}\delta b,c\frac{N_{A}}{2}\delta a-d\frac{N_{B}}{2}\delta b\right)\\ P_{21}: & \left(-\alpha \left(z\right)\frac{N_{A}}{2}\delta a+\beta \left(z\right)\frac{N_{B}}{2}\delta b,-c\frac{N_{A}}{2}\delta a+d\frac{N_{B}}{2}\delta b\right)\\ P_{22}: & \left(-\alpha \left(z\right)\frac{N_{A}}{2}\delta a-\beta \left(z\right)\frac{N_{B}}{2}\delta b,-c\frac{N_{A}}{2}\delta a-d\frac{N_{B}}{2}\delta b\right) \end{array},$$

where *c* and *d* are the coefficients of the second line of the matrix $B=A^{-1}$, that are arbitrary for refocusing. Thus, by defining $a_{z}$ and $b_{z}$ as the minimum and maximum of the *t* coordinates, one gets

$$b_{z}=\max\left\{\pm c\frac{N_{A}}{2}\delta a\pm d\frac{N_{B}}{2}\delta b\right\}=\left|c\right|\frac{N_{A}}{2}\delta a+\left|d\right|\frac{N_{B}}{2}\delta b$$

and

$$a_{z}=\min\left\{\pm c\frac{N_{A}}{2}\delta a\pm d\frac{N_{B}}{2}\delta b\right\}=-\left|c\right|\frac{N_{A}}{2}\delta a-\left|d\right|\frac{N_{B}}{2}\delta b,$$

which demonstrates that the maximum integration range is the one reported in Equation (14). Interestingly, the FOV of Equation (4) can be obtained by applying the same reasoning on the *x* coordinates.
